# Lentiviral and AAV-mediated expression of palivizumab offer protection against Respiratory Syncytial Virus infection

**DOI:** 10.1038/s41598-021-95150-z

**Published:** 2021-08-03

**Authors:** Agata Antepowicz, Omar Habib, Freja Kirsebom, Cecilia Johansson, Deborah R. Gill, Stephen C. Hyde

**Affiliations:** 1 Gene Medicine Research Group, NDCLS, Radcliffe Department of Medicine, John Radcliffe Hospital, University of Oxford, Oxford, UK; 2 Respiratory Infections, National Heart and Lung Institute, Imperial College London, London, UK

**Keywords:** Gene delivery, Gene therapy, Nucleic-acid therapeutics

## Abstract

Respiratory syncytial virus (RSV) infection is a common cause of hospitalisation in infants and the elderly. Palivizumab prophylaxis is the only approved treatment modality but is costly and only offered to select vulnerable populations. Here, we investigated gene delivery approaches via recombinant adeno-associated virus (rAAV2/8) and simian immunodeficiency virus (rSIV.F/HN) vectors to achieve sustained in vivo production of palivizumab in a murine model. Delivery of palivizumab-expressing vectors 28 days prior to RSV challenge resulted in complete protection from RSV-induced weight loss. This approach offers prophylaxis against RSV infection, allowing for wider use and reduction in treatment costs in vulnerable populations.

## Introduction

Respiratory syncytial virus (RSV) is the most common cause of acute lower respiratory tract infections in infants and young children. In 2015, global RSV infection in children younger than 5 years was estimated at 33.1 million, of which about 3.2 million required hospitalisation with 59,600 in-hospital deaths^1^. Infection with RSV in children is also associated with long-term complications, such as recurrent wheeze and potentially asthma^2^, rendering the virus a severe burden in the paediatric population. Other high-risk populations include the elderly, those with chronic lung or heart disease and the immunosuppressed^3^.

To date, no licensed RSV vaccine is available, and the only approved intervention is passive immunisation with the monoclonal antibody (mAb) palivizumab (Synagis®). To provide protection throughout the RSV season, multiple doses of palivizumab at monthly intervals are required to achieve sufficient mAb bioavailability. Due to the relatively high cost, estimated between £2500 and £4200 per person per season^4^, its use has been limited to select, vulnerable infant populations. Adopting gene therapy for passive immunisation via mAb gene transfer, also known as vectored-immunoprophylaxis^5,6^, could provide sustained in vivo mAb production, offering protection throughout the entire RSV season, making the treatment more widely accessible.

Here, we investigated two viral vector platforms to direct in vivo expression of palivizumab via either the intranasal or intramuscular route. The first is a recombinant lentiviral vector based on simian immunodeficiency virus (SIV) pseudotyped with Sendai virus envelope proteins F and HN (known as rSIV.F/HN)^7^. A single intranasal rSIV.F/HN dose directs sustained expression of α1-antitrypsin in both the lung lumen and systemic circulation of mice for at least 19 months^8^, highlighting this platform as a candidate for long-term expression of palivizumab. Recombinant adeno-associated virus serotype 8 (rAAV2/8) vector delivered intramuscularly also directs long-term expression of antibodies and other transgenes in both mice and man^9^. We hypothesised that these vectors would direct robust, long-term expression of palivizumab to protect animals against an RSV challenge.

## Results

### Generation of viral vector genomes

**Figure 1:**
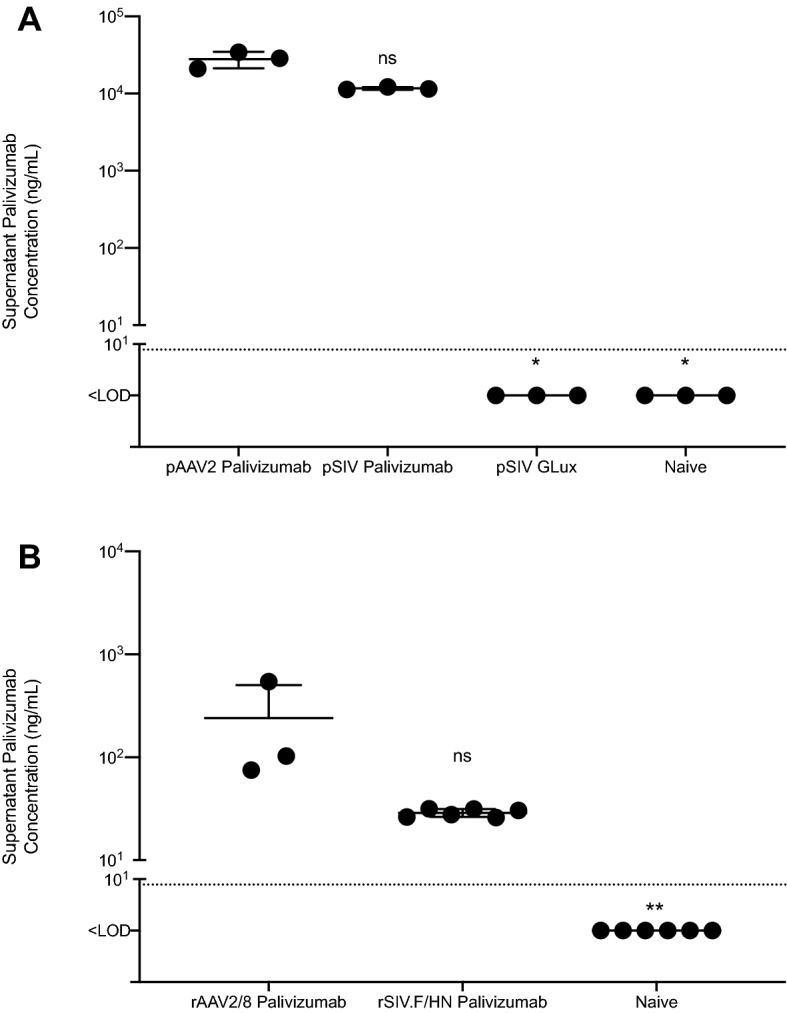
In vitro production of palivizumab from rAAV2/8 and rSIV.F/HN. HEK293T cells were (**A**) transfected with rAAV and rSIV vector genomes expressing palivizumab or GLux, were (**B**) transduced with rAAV and rSIV vector particles expressing palivizumab or remained naïve to treatment; 48 h post-transfection/transduction, palivizumab levels in tissue culture supernatant was measured using a Human IgG ELISA. In several cases the errors are obscured by the mean bar, where appropriate, only positive error bars are shown. The dotted line represents the limit of detection (LOD). Differences between treatment and naïve control groups were evaluated using the Kruskal–Wallis test with Dunn’s post hoc multiple comparison test.

A single-open reading frame (ORF) of palivizumab^10^, was constructed in which the light and heavy chain sequences were fused via a protease cleavage site that combined a 2A self-processing peptide and a furin target sequence^11^. To improve palivizumab expression, the transgene was codon-optimised for *Homo sapiens* and depleted of CpG dinucleotides. Vector genome plasmids for two palivizumab-expressing viral vectors, rAAV2 with muscle-active CASI promoter and rSIV with lung-active hCEF promoter, were generated. Similar vector genome plasmids where the palivizumab ORF was replaced with the ORF for *Gaussia* luciferase (GLux) were also created. Transfection of HEK293T cells with the palivizumab vector genome plasmids resulted in robust palivizumab protein expression (Fig. 1A). The rAAV2 vector genomes were used to produce rAAV2/8 serotype vector particles, and the rSIV vector genomes were used to produce rSIV.F/HN pseudotyped vector particles. Transduction of HEK293T cells with the palivizumab vector particles also resulted in robust palivizumab protein expression (Fig. 1B).

### In vivo reporter gene production using intramuscular rAAV2/8 and intranasal rSIV.F/HN delivery

**Figure 2:**
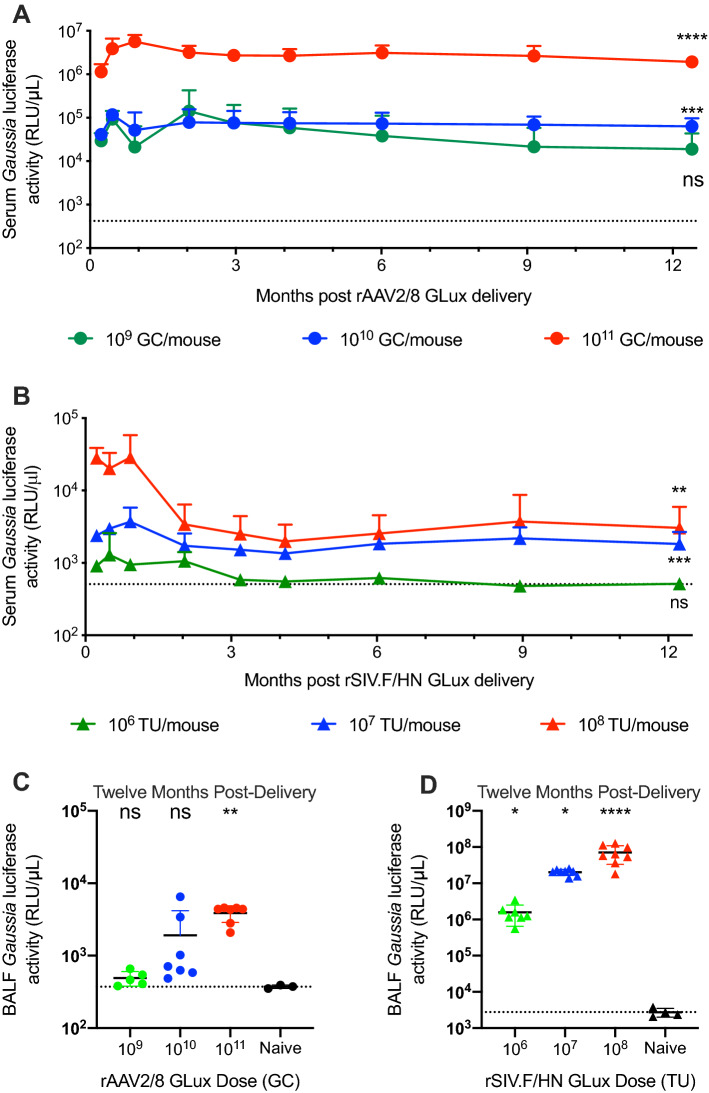
In vivo production of *Gaussia* Luciferase from rAAV2/8 and rSIV.F/HN (**A**) Female BALB/c mice were administered 10^9^ (green circles), 10^10^ (blue circles) or 10^11^ (red circles) Genome Copies (GC) of rAAV2/8 CASI Glux via intramuscular (IM) injection (n = 12/group) or were naïve to treatment (n = 22); or, (**B**) 10^6^ (green triangles), 10^7^ (blue triangles) or 10^8^ (red triangles) Transducing Units (TU) of rSIV.F/HN hCEF GLux via intranasal (IN) instillation (n = 11/group) or were naïve to treatment (n = 16). GLux activity was determined in serum was obtained via tail vein bleeding at the indicated time-points. Individual values for GLux activity in BALF samples from (**C**) rAAV2/8 and (**D**) rSIV.F/HN treatment groups was determined at the end of the study (approximately 12 months post vector delivery; n = 3–8/group from groups described in **A** & **B**). The dotted line represents the mean naïve value. Differences between treatment groups and naïve animals were evaluated using the Kruskal–Wallis test with Dunn’s post hoc multiple comparison test.

As a simple surrogate for palivizumab expression, we first assessed the activity of the GLux vector particles to direct expression of GLux protein after in vivo delivery. Mice were administered three ascending, single doses of rAAV2/8 GLux vector via intramuscular (IM) delivery, or rSIV.F/HN GLux vector via intranasal (IN) delivery. Mid and high doses (10^10^ or 10^11^ Genome Copies (GC)) of rAAV2/8 GLux resulted in abundant serum GLux activity, detectable from as early as day 7, which was sustained for at least 12 months at 63.5e3 ± 33.3e3 and 1.9e6 ± 0.6e6 RLU/µL, respectively (*p* = 0.0009 and *p* < 0.0001) (Fig. 2A). The low rAAV2/8 GLux dose (10^9^ GC) also tended to direct detectable GLux activity (18.9e3 ± 24.8e3 RLU/µL), but this level was not significantly different from animals naïve to treatment (419 ± 142 RLU/µL; *p* = 0.0745). Serum levels achieved at 12 months with the mid and high doses (10^7^ or 10^8^ Transducing Units (TU)) of the rSIV.F/HN GLux vector (1.8e3 ± 0.8e3 or 3.0e3 ± 2.9e3 RLU/µL) were significantly higher than naïve animals (*p* = 0.0004 and *p* = 0.0031 respectively) (Fig. 2B), but markedly lower than those achieved with rAAV GLux vectors. As with rAAV expressing GLux, serum levels with the low dose of rSIV.F/HN GLux (1e6 TU; 514 ± 75 RLU/µL) were not significantly different from naïve animals (*p* > 0.9999).

We were also interested in the expression levels achieved in bronchoalveolar lavage fluid (BALF), a potentially more relevant sample than serum as it represents the fluid space in which RSV infections occur. While levels of serum GLux achieved after rAAV2/8 IM vector delivery (Fig. 2A) eclipsed the levels achieved after rSIV.F/HN IN vector delivery (Fig. 2B), the profile of GLux activity in BALF was reversed, such that peak expression levels achieved with rAAV2/8 GLux (3.9e3 ± 1.0e3 RLU/µL) (Fig. 2C) were markedly lower than the peak levels achieved with rSIV.F/HN vectors, which ranged up to 71e6 ± 38e6 RLU/µL (naïve 2.7e3 ± 0.8e3 RLU/µL) (Fig. 2D).

### In vivo palivizumab production using intramuscular rAAV2/8 and intranasal rSIV.F/HN delivery

**Figure 3:**
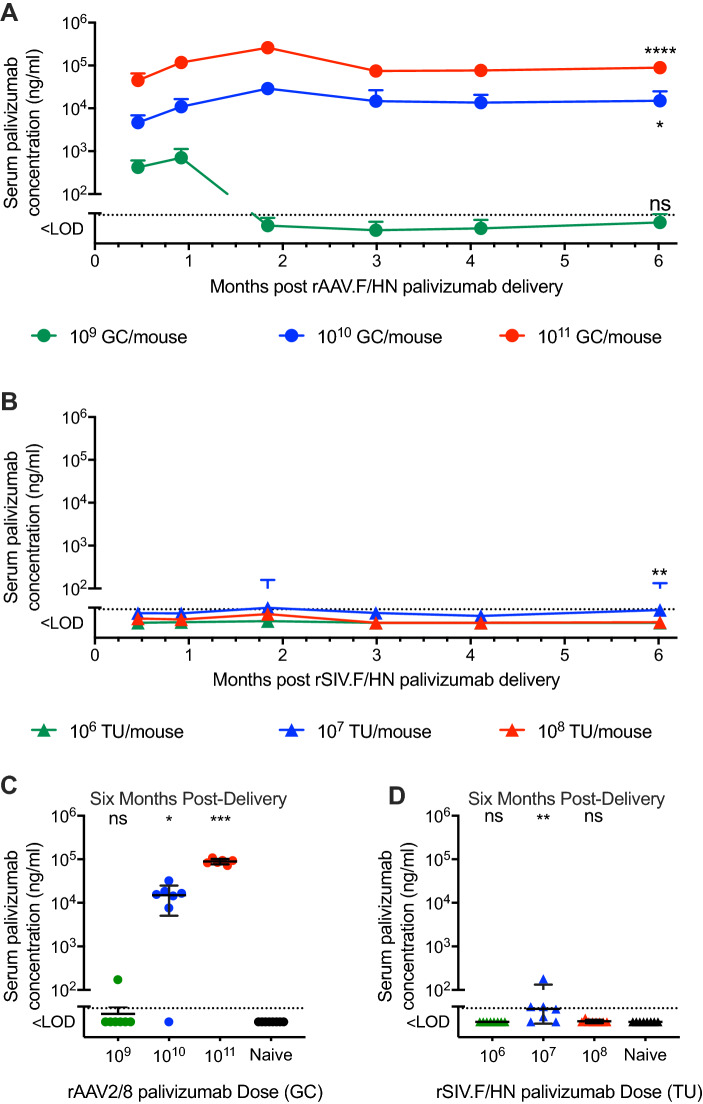
In vivo production of palivizumab from rAAV2/8 and rSIV.F/HN: serum. (**A**) Female BALB/c mice were administered 10^9^ (green circles), 10^10^ (blue circles) or 10^11^ (red circles) Genome Copies (GC) of rAAV2/8 CASI palivizumab via intramuscular (IM) injection, were naïve to treatment; or (**B**) 10^6^ (green triangles), 10^7^ (blue triangles) or 10^8^ (red triangles) Transducing Units (TU) of rSIV.F/HN hCEF palivizumab via intranasal (IN) instillation (n = 16/group). Serum was obtained via tail vein bleeding at the indicated time-points and palivizumab levels determined using a human IgG ELISA. Individual values for serum palivizumab levels from (**C**) rAAV2/8 and (**D**) rSIV.F/HN treatment groups was determined at the end of the study (approximately 6 months post vector delivery; n = 6–8/group from groups described in **A** & **B**). The dotted lines represent the mean naïve value. Differences between treatment groups and naïve animals were evaluated using the Kruskal–Wallis test with Dunn’s post hoc multiple comparison test.

These results established that robust, long-lasting, in vivo expression could be achieved for a simple reporter protein using our rAAV2/8 and rSIV.F/HN vector systems and we next evaluated in vivo palivizumab expression. Mice were administered three ascending, single doses of rAAV2/8 palivizumab vector via IM delivery, or rSIV.F/HN vector via IN delivery and serum palivizumab levels were evaluated over 6 months post-delivery (Fig. 3A, B respectively). Mid and high doses (10^10^ or 10^11^ Genome Copies) of rAAV2/8 resulted in marked serum palivizumab, detectable as early as day 14 (Fig. 3A), which were sustained for at least 6 months at 15.0 ± 9.9 and 89.3 ± 12.3 µg/mL, respectively (*p* = 0.0356 and *p* < 0.0001) (Fig. 3A and C). The low rAAV2/8 dose (10^9^ GC) was undetectable after day 28. As anticipated, only marginal levels of palivizumab were detected in the serum at any dose of rSIV.F/HN (Fig. 3B and D) since IN delivery of this vector typically results in higher transgene levels in the lungs compared with systemic circulation^8,12^. Individual palivizumab levels achieved at 6 months post rSIV.F/HN delivery illustrate the minimal levels observed compared with those achieved after rAAV2/8 delivery (Fig. 3D).

**Figure 4:**
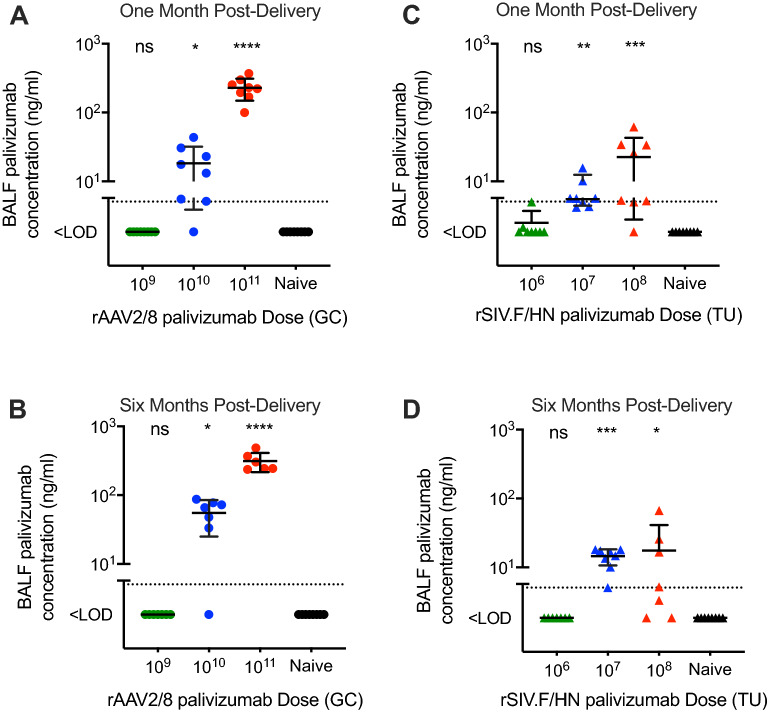
In vivo production of palivizumab from rAAV2/8 and rSIV.F/HN: BALF. Female BALB/c mice (n = 16/group) were administered 10^9^ (green circles), 10^10^ (blue circles) or 10^11^ (red circles) Genome Copies (GC) of rAAV2/8 CASI Glux via intramuscular (IM) injection; 10^6^ (green triangles), 10^7^ (blue triangles) or 10^8^ (red triangles) Transducing Units (TU) of rSIV.F/HN hCEF palivizumab via intranasal (IN) instillation, or were naïve to treatment. Animals treated with (**A** and **B**) rAAV2/8 or (**C** and **D**) rSIV.F/HN were culled at either 1 month (**A** and **C**) (n = 8/group), or 6 months (**B** and **D**) (n = 6–8 group) post-delivery to obtain BALF. Palivizumab levels in the BALF was measured using a Human IgG ELISA. The dotted line represents the LOD. Differences between treatment groups and naïve animals were evaluated using the Kruskal–Wallis test with Dunn’s post hoc multiple comparison test.

Significant palivizumab expression was observed in the bronchoalveolar lavage fluid (BALF) of mice administered mid and high (10^10^ and 10^11^ GC) doses of rAAV2/8, with palivizumab levels reaching 18.3 ± 13.8 and 230 ± 81.7 ng/mL at 1 month (Fig. 4A; *p* = 0.0456 and *p* < 0.0001). This expression was sustained for the duration of the study with comparable palivizumab levels of 55.2 ± 30.1 and 313.6 ± 98.2 ng/mL at 6 months (Fig. 4B; *p* = 0.0423 and *p* < 0.0001). In mice administered mid and high (10^7^ and 10^8^ TU) doses of rSIV.F/HN, palivizumab levels in the BALF reached 9.2 ± 3.2 and 22.6 ± 20.3 ng/mL) at 1 month (Fig. 4C; *p* = 0.0018 and *p* = 0.0007), sustained for the duration of the experiment, averaging 14.5 ± 3.8 and 17.6 ± 23.6 ng/mL respectively, in the BALF at 6 months (Fig. 4D; *p* = 0.0006 and *p* = 0.0228). Using an approximate conversion technique^8^, the epithelial lining fluid (ELF) levels of the palivizumab at 6 months post-delivery was estimated as 9 µg/mL for rAAV2/8 and 0.5 µg/mL for rSIV.F/HN at the highest doses. The results indicate these vector platforms can provide robust palivizumab expression in vivo.

### Protection from weight loss in a mouse model of RSV infection

**Figure 5:**
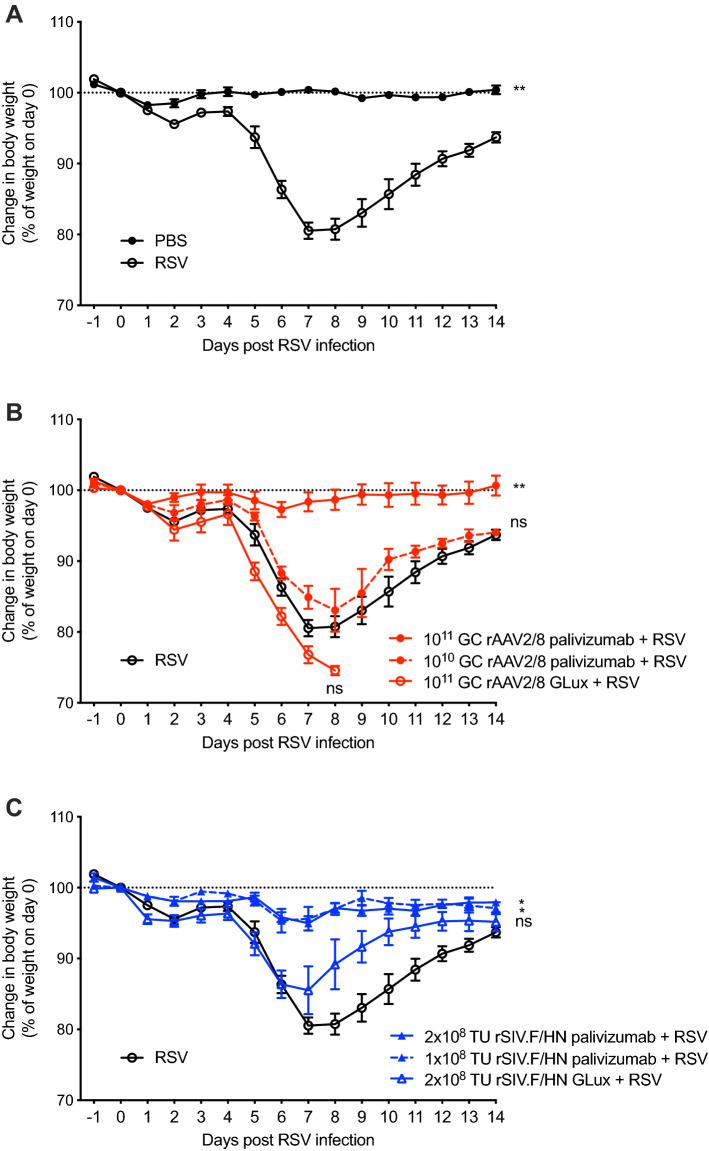
Protection against RSV infection with vector-mediated palivizumab gene transfer. (**A**) Female BALB/c mice (n = 5/group) were infected with 7 × 10^5^ FFU of RSV via IN instillation or sham infected with PBS and their percentage weight change was recorded for 14 days. (**B**) Female BALB/c mice (n = 5/group) were administered rAAV2/8 vector expressing palivizumab (10^10^ or 10^11^ GC), or GLux (10^11^ GC) via IM injection: or (**C**) rSIV.F/HN vector expressing palivizumab (10^8^ or 2 × 10^8^ TTU) or GLux (2 × 10^8^ TTU) via IN instillation. Twenty-eight days later, mice were infected with 7 × 10^5^ FFU of RSV via IN instillation and their percentage weight was recorded for 14 days. For clarity, symbols and error bars represent mean ± standard error of the mean. Missing weight data after day 7 for the rAAV2/8 GLux dosed mice is due to the application of a pre-determined humane endpoint in this treatment group. The area under the weight loss curve up to day 7 was calculated for each animal and the differences between treatment groups and RSV challenged animals were evaluated using the Kruskal–Wallis test with Dunn’s post hoc multiple comparison test.

In our mouse model of RSV infection, animals challenged with 7 × 10^5^ focus-forming units (FFU) of RSV delivered IN experienced pronounced weight loss over a ~ 7 day period. In the majority of animals, weight loss was reversible and was recovered over the next ~ 7 days (Fig. 5A). Animals losing > 20% of their initial body weight for more than 2 days are humanely euthanised, to limit the severity of their experience.

We hypothesised that in vivo palivizumab expression by rAAV8 and rSIV.F/HN vectors would protect mice against RSV-induced weight loss. Mice were therefore administered a single dose of vector expressing palivizumab, either rAAV2/8 via the IM route (10^10^ or 10^11^ GC) (Fig. 5B) or rSIV.F/HN (10^8^ or as an increased dose had proven informative in similar protection studies following influenza virus challenge^12^, the maximal feasible dose 2 × 10^8^ TTU) via the IN route (Fig. 5C). Two groups received matched high doses of vectors expressing control GLux reporter gene. Twenty-eight days post-dosing, mice were challenged with RSV as described above. Mice dosed with either rAAV2/8 or rSIV.F/HN expressing GLux were not protected from weight loss (*p* = 0.5986 and *p* > 0.9999; Fig. 5B and C respectively). In contrast, complete protection from RSV-induced weight loss was achieved in mice administered the highest dose of rAAV2/8 (*p* = 0.0032; Fig. 5B), as well as both doses of rSIV.F/HN, expressing palivizumab (*p* = 0.0159 and *p* = 0.0159 respectively; Fig. 5C). Interestingly, mice dosed with 10^10^ GC of rAAV2/8 expressing palivizumab were not protected from weight loss (*p* > 0.9999; Fig. 5B). All animals survived the study except for those receiving 10^11^ GC of rAAV2/8 expressing GLux prior to RSV challenge; these mice had more pronounced RSV-induced weight loss than any other treatment group including mice that only received RSV challenge (*p* < 0.0079). As these mice experienced > 20% RSV-induced weight loss, they were humanely euthanised at day 8 post RSV challenge (Fig. 5B).

**Figure 6:**
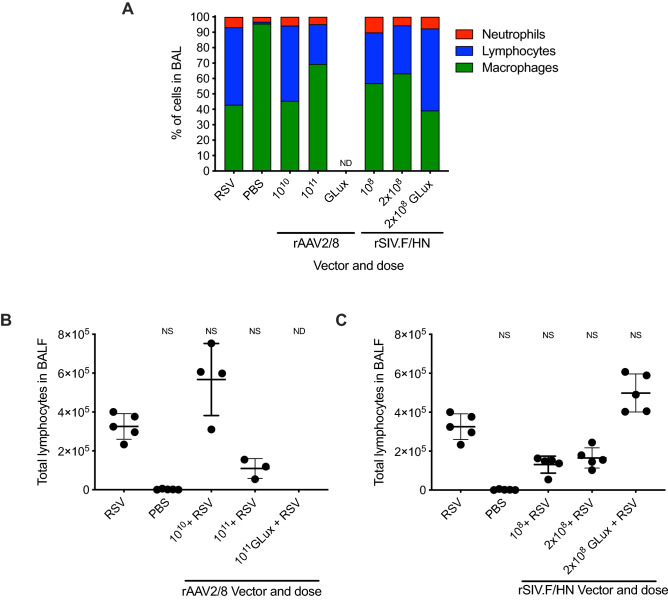
Changes in lung leukocyte cell populations following vector-mediated palivizumab gene transfer and subsequent RSV infection. Female BALB/c mice (n = 5/group) were administered rAAV2/8 vector expressing palivizumab (10^10^ or 10^11^ GC), or GLux (10^11^ GC) via IM injection, or rSIV.F/HN vector expressing palivizumab (10^8^ or 2 × 10^8^ TTU) or GLux (2 × 10^8^ TTU) via IN instillation. Twenty-eight days later, mice were infected with 7 × 10^5^ FFU of RSV via IN instillation. Differential leukocyte quantification was carried out on H&E stained cytospin slides prepared from BALF cells recovered 14 days post RSV or PBS challenge; (**A**) the relative proportions of each cell type, and, the absolute numbers of lymphocytes following (**B**) rAAV2/8-mediated or (**C**) rSIV.F/HN-mediated palivizumab or GLux expression are shown. Missing leukocyte (ND: **A** & **B**) data for rAAV2/8 GLux dosed mice is due to the application of a pre-determined humane endpoint in this treatment group. Differences between the treatment groups and RSV challenged animals were evaluated using the Kruskal–Wallis test with Dunn’s post hoc multiple comparison test.

We also evaluated immune cell recruitment into the airways following RSV infection in these animals. The relative proportion of macrophages, lymphocytes and neutrophils as well as the absolute numbers of lymphocytes in the BALF were determined at the end of the study (Fig. 6A–C). As expected, lymphocytes were increased by exposure to RSV (*p* = 0.0183)^13^. There was a dose-dependent trend for palivizumab expression with both rAAV2/8 (Fig. 6B) and rSIV.F/HN (Fig. 6C) to reduce RSV-associated lymphocytes, but this result did not reach significance due to lack of statistical power. Overall, these results indicate that vector-mediated expression of palivizumab in mice can help protect against weight loss due to RSV infection.

## Discussion

In this study we sought to evaluate the ability of two different gene transfer vectors systems to direct long-term, prophylactic expression of a mAb to inhibit lung infection by RSV. We chose to evaluate the rAAV2/8 and rSIV.F/HN vector systems as they have alternate preferred delivery routes, and they have also been used by us and others to establish in vivo expression of other therapeutic proteins^6,7,12^.

Here, we first confirmed that both rAAV2/8, via IM injection, and rSIV.F/HN, via IN delivery, could direct long-term expression of GLux, a common reporter gene. While dose-dependent serum GLux expression was achieved with both vector systems, the peak levels achieved with rAAV2/8 were approximately 100-fold higher than achieved with rSIV.F/HN. After rAAV2/8 vector delivery, serum Glux activity rose to a plateau after approximately 30 days and was sustained essentially unchanged for at least 1 year. Sustained GLux expression was also seen after rSIV.F/HN vector delivery although the kinetics were somewhat different; with GLux levels falling from a peak after approximately 30 days post-delivery to a plateau after approximately 60 days which was then sustained essentially unchanged for at least 1 year. GLux activity was also measured in the BALF, a sample representing the fluid lining the lung epithelium that is the site of RSV infection. At 1 year after vector delivery GLux activity was approximately 10,000-fold higher with the rSIV.F/HN vector system, suggesting that local expression within the lung allowed GLux protein accumulation.

Subsequently, we established that both vector systems could direct expression of palivizumab, a prototype anti-RSV mAb, after both in vitro and in vivo transduction. As with the GLux reporter protein, palivizumab serum levels were highest after rAAV2/8 delivery. In contrast to the results with the GLux reporter protein, BALF palivizumab levels were approximately tenfold higher with the rAAV2/8 than the rSIV.F/HN vector system. Together, these data suggest that GLux is not necessarily a representative reporter protein when developing therapeutic proteins with IgG properties. Importantly, active transport processes exist to aid selective redistribution of immune complexes^14^ an advantage that the GLux reporter may lack. Nevertheless, both vectors demonstrated significant palivizumab expression in BALF for at least 6 months post-delivery—the duration of the study.

Finally, both vector systems directed sufficient palivizumab expression such that at the higher end of the dose ranges used, both vectors offered complete protection from the weight loss observed in our RSV challenge model. This suggests that clinical benefit could be offered by translating this novel treatment modality to at-risk human populations. Interestingly, in these challenge studies, treatment with rAAV2/8 GLux vector appeared to increase the weight loss experienced after RSV infection, while rSIV.F/HN GLux vector treatment appeared to have no effect. This might be indicative of some perturbation in physiology induced by either the rAAV2/8 vector system itself, or to high serum levels of the GLux reporter protein. Further studies are perhaps warranted to more fully understand this phenomenon prior to development of rAAV2/8 -based therapeutics.

The kinetics of palivizumab expression with both vector systems following a single vector administration are consistent with protection for a complete 31-week RSV season, an improvement over passive vaccination with palivizumab protein, which is typically repeated monthly. Intriguingly, transgene expression from both vectors has been observed for the life-time of experimental animals, suggesting that protection in humans could reasonably be expected to extend into subsequent RSV seasons, a distinct advantage in the context of vulnerable populations suffering repeated RSV infections^3^. In the studies presented here, protection against RSV challenge was only determined at a single time-point, 4 weeks after palivizumab vector delivery; further studies are warranted to establish that the duration of RSV protection matches the longevity of palivizumab expression observed.

Long-term in vivo expression of an IgG such as palivizumab via the antibody gene transfer approaches described is not anticipated to be in any way problematic or deleterious. The maximum serum levels achieved in this study (~ 100 µg/mL following rAAV2/8 delivery) are approximately × 100 lower than typical adult circulating IgG levels^15^, and thus non-specific effects of the expressed palivizumab seem highly unlikely. Furthermore, the safety and efficacy of palivizumab after bolus protein delivery (which results in peak levels of a similar magnitude) is well established^16^. Importantly, a clinical study of a similar rAAV2/8 in vivo delivery approach for a broadly neutralising anti-HIV antibody shows the approach is safe, and directs persistent antibody expression for at least ~ 2 years post-delivery^17^. Conversely, specific anti-transgene antibodies (ATA) could confound the chosen approach. In clinical gene transfer studies with rAAV, anti-transgene immune responses have been largely reported where the intramuscular delivery route has been used, leading to the suggestion that the use of this organ as a depot for protein production may favour the generation of ATA^18^. As a precaution, we included miR-142-3p target sites in both our rAAV and rSIV vector genomes in the anticipation that this would minimise transgene expression in antigen-presenting cells and limit the generation of ATA^19^.

The antibody gene transfer approach outlined here is an uncommon approach to achieving passive immunity. The vector systems utilised to achieve antibody expression have in themselves some noteworthy properties. The rSIV.F/HN lentiviral vector system used is expected to integrate its vector sequences into the host genome. This genome integration property is common to retrovirus-derived vector systems and has proven to be problematic for vectors derived from Moloney murine leukaemia virus (MMLV). In early clinical studies with MMLV vectors, integration close to proto-oncogene transcriptional start sites led to unwanted overexpression of factors that drove uncontrollable cellular division^20^. Lentiviral vectors, such as rSIV.F/HN, show altered genomic integration preferences—essentially integrating at random within transcribed regions, a feature that favours integration in intronic sequences away from transcriptional start sites^21^. It is reassuring, that despite exposure to a vastly higher number of research subjects, lentiviral vectors have yet to be associated with similar clinical genotoxicity^22–25^. In contrast, rAAV vector genomes largely remain extrachromosomal and thus unlike lentiviral vectors, their perceived genotoxicity risk is negligible; there is however, emerging evidence that under certain circumstances rAAV-dependent genotoxicty can occur^26,27^.

While the degree of protection offered by both vectors was similar, rAAV2/8 directed higher levels of palivizumab both in the serum and BALF, and offered an easily translatable IM delivery route. In contrast, the rSIV.F/HN platform requires inhalation, which might be challenging in the context of a child with a history of respiratory disorders. However, the rSIV.F/HN vector has a crucial advantage, the ability to offer repeated efficacy after at least three doses^28,29^, which has proven difficult to achieve with a single serotype of rAAV in the lung^30^. We believe this ability offers the rSIV.F/HN platform a crucial translational advantage. The emerging popularity of rAAV-vectors for in vivo gene transfer, and thus immunological restriction to subsequent vector use, could provide a new challenge to the field. Encouragingly, efforts to reduce this immunological restriction via transient rAAV-specific IgG removal^31^ or destruction^32,33^ appear under certain circumstances to overcome this barrier.

Further studies are warranted prior to clinical development including more prolonged evaluation of the duration of effect and the ability for this effect to be replicated in higher-order model systems—likely including non-human primates. One significant barrier to widespread acceptance of this approach will be the cost of goods. Gene therapy products tend to attract high value pricing, not least because they tend to be developed for niche rather than high-volume markets. We note that the COVID-19 pandemic has resulted in the speedy development of multiple vaccines which can also be thought of as gene therapy vectors. For example, mRNA/lipid vaccines are exemplars of non-viral vector gene transfer, while adenoviral vector vaccines are examples of viral vector gene transfer. We speculate that the investment in manufacturing and storage infrastructure necessary for such therapeutics may help reduce cost of goods for conventional gene therapies and simplify regulatory pathways to drug licensing.

In conclusion, we demonstrate here that gene transfer, mediated by rAAV2/8 and rSIV.F/HN, can direct therapeutically relevant levels of palivizumab in the murine circulation and lung lumen, which is protective against RSV infection.

## Methods

### Lentiviral vector production and titration

rSIV.F/HN lentiviral vector particles were produced essentially as described previously^8^, via a five-plasmid transient transfection method using HEK293T cells grown in suspension. Briefly, the single ORF cDNA of palivizumab was configured using publicly available sequence^10^ as described previously^11^, and CpG-depleted, human codon-optimized, and synthesized using the GeneArt Gene Synthesis service (Thermo Fisher Scientific). The palivizumab, or a CpG-free Gaussia Luciferase (soGLux), cDNA was inserted via unique NheI and PsPOMI sites into a recombinant simian immunodeficiency virus (SIV) genome plasmid backbone under the transcriptional control of the hCEF promoter^34^. Vectors were purified using anion exchange chromatography and tangential flow filtration and formulated into either FreeStyle293 media or TSSM buffer (tromethamine 20 mM, NaCl 100 mM, sucrose 10 mg/mL, and mannitol 10 mg/mL). The functional titre in Transducing Units per mL (TU/mL) of lentiviral vectors was determined based on the genomic integration of WPRE DNA sequence after transduction of HEK293F cells in vitro.

### rAAV vector production and titration

Recombinant rAAV was produced as described^35^. Briefly, the CpG-free palivizumab and Gaussia Luciferase (soGLux) cDNAs were inserted, via unique NheI and PsPOMI site, into a recombinant adeno-associated virus (AAV) genome plasmid backbone with AAV2 ITRs under the transcriptional control of the CASI promoter^6^. HEK293T cells were transfected with the plasmids pAdDeltaF6, pAAVRep2/Cap8, and prAAV2ITR with transgene using polyethylenimine (PEI; Polysciences Inc.). After 72 h, cells were resuspended in lysis buffer (1 M Tris(hydroxymethyl)aminomethane, 150 mM NaCl) and EDTA-free protease inhibitor cocktail and underwent four freeze–thaw cycles. Cell lysates were incubated (37ºC for 30 min) with Benzonase (50 U/mL final concentration) and clarified via centrifugation, and purified using iodixanol gradient fractionation and diafiltration into D-PBS using Amicon Ultra-15 100 K MWCO filters. The number of Genome Copies (GC/mL) was determined by qPCR.

### Animal studies

All procedures involving laboratory mice were carried out in accordance with UK Home Office approved project and personal licenses under the terms of the Animals (Scientific Procedures) Act 1986 (ASPA 1986), were approved by the University of Oxford or Imperial College Animal Welfare Ethical Review Body as appropriate, and are reported in compliance with the ARRIVE guidelines (https://arriveguidelines.org). Female BALB/c mice, 6–8 weeks old at the initiation of studies, were used. Animals were arbitrarily assigned to study groups using an open-label randomised block approach. Overall, 259 animals were used (Fig. 2A: n = 12/rAAV2/8 group, 3 groups, n = 22 naïve. Figure 2B: n = 11/rSIV.F/HN group, 3 groups, n = 16 naive. Figure 2C, D animals (n = 3–8/group) from Fig. 2A, B respectively. Figure 3A, B: n = 16/group, Fig. 3C, D animals (n = 6–8/group) from Fig. 3A, B respectively. Figure 4A–D: animals (n = 6–8/group) from Fig. 3. Figure 5: n = 5/group, 8 groups. Figure 6: animals (n = 3–5/group) from Fig. 5). Group sizes reduced during the study as effect sizes became more predictable. Animals were euthanised at the end of each study by cervical dislocation or intraperitoneal injection of 200 mg/kg pentobarbital.

### Administration of vector and virus to mice

Anaesthesia was induced using inhalation of 4–4.5% isoflurane (Abbott, Maidenhead, UK) and maintained with 2.5–3.5% isofluorane. For delivery of lentiviral vector and for RSV infection, a total volume of 100 μL was administered by nasal sniffing as previously described^36^ Adeno-associated viral vector was administered by injection into the gastrocnemius or quadriceps muscle using a 500 μL insulin syringe with 29G needle in a total volume of 40 μL.

### Collection of samples from mice

Blood was collected from the tail vein of mice, stored overnight at 4 °C and centrifuged to isolate serum. For collection of broncho-alveolar lavage fluid (BALF), mice were euthanised and dissected to expose the trachea. A small tear was made in the trachea and a 0.75 mm cannula used to infuse the lung with 1 mL BALF solution (PBS, 50 µM EDTA; 1% BSA was included except for leukocyte quantification) and then fluid collected by gentle aspiration. Flushing the lung was performed three times using the same (for studies with monoclonal antibodies), or fresh (for studies with GLux) BALF solution. For quantification of protein expression, the collected BALF was centrifuged to sediment cells, and the supernatant used for ELISA or luciferase assay as described below.

### Quantification of protein expression

Palivizumab levels in the cell culture media, serum and BALF were measured using Human IgG ELISA kit (Bethyl, Cambridge, UK) according to the manufacturer’s instructions. GLux activity in the serum was measured using BioLux *Gaussia* Luciferase Assay Kit (NEB, Ipswich, USA) according to the manufacturer’s instructions. Expression levels in ELF were corrected for the dilution using lavage fluid collection urea assay as described previously^8^.

### Total and differential leukocyte quantification

The BALF was centrifuged at 3,500 rcf and the cell pellet resuspended in Ammonium-Chloride-Potassium buffer (150 mM NH4Cl, 10 mM KHCO3, 0.1 mM EDTA) to lyse red blood cells. After 2 min, DMEM (Sigma-Aldrich) was added, the samples were centrifuged at 3,500 rcf and the pellet was resuspended in 500 µl of DMEM. For total leukocyte quantification, an aliquot of the cell suspension was mixed with 0.1% trypan blue and cells manually counted using a haemocytometer. For differential leukocyte quantification, 100 µL of the cell suspension was transferred onto a glass slide (Tharmac, Waldsoms, Germany) using Cytospin III Cytocentrifuge (Thermo Fisher Scientific) and allowed to air-dry. The slides were fixed and stained using Reastain Quick-Diff Kit (Reagena, Toivala, Finland) and visualised using a light microscope. At least 300 cells per slide were counted, differentiating between macrophages, lymphocytes and neutrophils based on their morphology.

### Statistics

Group sizes were initially projected from previous data^12^, using IgG expression and RSV-induced weight loss as the primary endpoints and were reduced throughout the study as estimates of effect size became more robust. G*Power software v3.1.9.6^37^ was used to project group sizes and establish achieved power. It proved impossible to recover BALF from all animals, this was expected as the procedure is technically challenging, resulting in missing datapoints in some BALF related assays. This was anticipated, and group sizes were adjusted a priori to ensure robust statistical analyses were possible. Statistical analysis was performed using Prism software v8.4.2 for Mac (GraphPad Software). As normality of data distribution could not be assumed with the refined group sizes selected for several of the animal studies, the non-parametric Kruskal–Wallis test with Dunn’s multiple comparisons post-hoc test was used to compare experimental groups with the indicated negative control group throughout. For weight loss studies, area under the curve values for the 7 days post RSV challenge were first calculated for each animal prior to Kruskal–Wallis evaluation. Fisher’s exact contingency analysis test was used to evaluate the proportion of mice escaping the humane endpoint after RSV challenge. Numerical values in text are presented as mean ± standard deviation. Symbols and error bars in figures represent the mean and standard deviation unless stated otherwise (see Fig. 5). In some cases, error bars are obscured by mean symbols. In some cases where a logarithmic y-axis is used, only positive error bars are shown. A calculated *p*-value of *p* < 0.05 was deemed a significant difference and indicated on figures where appropriate. In figures, the symbols ns, *, **, *** and **** represent *p* > 0.05, < 0.05, *p* < 0.01, *p* < 0.001 and *p* < 0.0001 respectively.

## References

[1] Shi T, McAllister DA, O'Brien KL, Simoes EAF, Madhi SA, Gessner BD (2017). Global, regional, and national disease burden estimates of acute lower respiratory infections due to respiratory syncytial virus in young children in 2015: a systematic review and modelling study. Lancet.

[2] Wu P, Hartert TV (2011). Evidence for a causal relationship between respiratory syncytial virus infection and asthma. Exp. Rev. Anti. Infect. Ther..

[3] Rose EB, Wheatley A, Langley G, Gerber S, Haynes A (2018). Respiratory syncytial virus seasonality—United States, 2014–2017. MMWR Morb. Mortal. Wkly. Rep..

[4] Nuijten MJ, Wittenberg W (2010). Cost effectiveness of palivizumab in Spain: an analysis using observational data. Eur. J. Health Econ..

[5] Skaricic D, Traube C, De B, Joh J, Boyer J, Crystal RG (2008). Genetic delivery of an anti-RSV antibody to protect against pulmonary infection with RSV. Virology.

[6] Balazs AB, Chen J, Hong CM, Rao DS, Yang L, Baltimore D (2011). Antibody-based protection against HIV infection by vectored immunoprophylaxis. Nature.

[7] Alton EW, Beekman JM, Boyd AC, Brand J, Carlon MS, Connolly MM (2017). Preparation for a first-in-man lentivirus trial in patients with cystic fibrosis. Thorax.

[8] Paul-Smith MC, Pytel KM, Gelinas JF, McIntosh J, Pringle I, Davies L (2018). The murine lung as a factory to produce secreted intrapulmonary and circulatory proteins. Gene Ther..

[9] Lin A, Balazs AB (2018). Adeno-associated virus gene delivery of broadly neutralizing antibodies as prevention and therapy against HIV-1. Retrovirology.

[10] Johnson S, Oliver C, Prince GA, Hemming VG, Pfarr DS, Wang SC (1997). Development of a humanized monoclonal antibody (MEDI-493) with potent in vitro and in vivo activity against respiratory syncytial virus. J. Infect. Dis..

[11] Fang J, Qian JJ, Yi S, Harding TC, Tu GH, VanRoey M (2005). Stable antibody expression at therapeutic levels using the 2A peptide. Nat. Biotechnol..

[12] Tan TK, Gamlen TPE, Rijal P, Townsend AR, Gill DR, Hyde SC (2020). Lung-targeting lentiviral vector for passive immunisation against influenza. Thorax.

[13] Kirsebom F, Michalaki C, Agueda-Oyarzabal M, Johansson C (2020). Neutrophils do not impact viral load or the peak of disease severity during RSV infection. Sci. Rep..

[14] Turula H, and Wobus CE. The Role of the Polymeric Immunoglobulin Receptor and Secretory Immunoglobulins during Mucosal Infection and Immunity. *Viruses.* 2018;10(5). 10.3390/v10050237 PMC5977230 29751532

[15] Agarwal S, Cunningham-Rundles C (2007). Assessment and clinical interpretation of reduced IgG values. Ann. Allergy Asthma Immunol..

[16] Forbes ML, Kumar VR, Yogev R, Wu X, Robbie GJ, Ambrose CS (2014). Serum palivizumab level is associated with decreased severity of respiratory syncytial virus disease in high-risk infants. Hum. Vaccin. Immunother..

[17] Casazza JP, Narpala S, Novik L, Yamshchikov G, Cale E, Doria-Rose N (2020). Durable HIV-1 antibody production in humans after AAV8-mediated gene transfer. Conf. Retroviruses Opportunistic Infect..

[18] Ronzitti G, Gross DA, Mingozzi F (2020). Human Immune Responses to Adeno-Associated Virus (AAV) Vectors. Front Immunol..

[19] Brown BD, Venneri MA, Zingale A, Sergi Sergi L, Naldini L (2006). Endogenous microRNA regulation suppresses transgene expression in hematopoietic lineages and enables stable gene transfer. Nat. Med..

[20] Hacein-Bey-Abina S, Von Kalle C, Schmidt M, McCormack MP, Wulffraat N, Leboulch P (2003). LMO2-associated clonal T cell proliferation in two patients after gene therapy for SCID-X1. Science.

[21] van Haasteren J, Munis AM, Gill DR, and Hyde SC. Genome-wide integration site detection using Cas9 enriched amplification-free long-range sequencing. *Nucleic Acids Res.* **49**(3), e16 (2021). 10.1093/nar/gkaa1152 PMC7897500 33290561

[22] Charrier S, Lagresle-Peyrou C, Poletti V, Rothe M, Cedrone G, Gjata B (2019). Biosafety studies of a clinically applicable lentiviral vector for the gene therapy of artemis-SCID. Mol. Ther. Methods Clin. Dev..

[23] Ellison SM, Liao A, Wood S, Taylor J, Youshani AS, Rowlston S (2019). Pre-clinical safety and efficacy of lentiviral vector-mediated ex vivo stem cell gene therapy for the treatment of mucopolysaccharidosis IIIA. Mol. Ther. Methods Clin. Dev..

[24] Grigor EJM, Fergusson D, Kekre N, Montroy J, Atkins H, Seftel MD (2019). Risks and benefits of chimeric antigen receptor T-cell (CAR-T) therapy in cancer: a systematic review and meta-analysis. Transfus Med. Rev..

[25] Rio P, Navarro S, Wang W, Sanchez-Dominguez R, Pujol RM, Segovia JC (2019). Successful engraftment of gene-corrected hematopoietic stem cells in non-conditioned patients with Fanconi anemia. Nat. Med..

[26] Li Y, Miller CA, Shea LK, Jiang X, Guzman MA, Chandler RJ (2021). Enhanced efficacy and increased long-term toxicity of CNS-directed, AAV-based combination therapy for krabbe disease. Mol. Ther..

[27] Nguyen GN, Everett JK, Kafle S, Roche AM, Raymond HE, Leiby J (2021). A long-term study of AAV gene therapy in dogs with hemophilia A identifies clonal expansions of transduced liver cells. Nat. Biotechnol..

[28] Mitomo K, Griesenbach U, Inoue M, Somerton L, Meng C, Akiba E (2010). Toward gene therapy for cystic fibrosis using a lentivirus pseudotyped with Sendai virus envelopes. Mol. Ther..

[29] Griesenbach U, Inoue M, Meng C, Farley R, Chan M, Newman NK (2012). Assessment of F/HN-pseudotyped lentivirus as a clinically relevant vector for lung gene therapy. Am. J. Respir. Crit. Care Med..

[30] Sumner-Jones SG, Gill DR, Hyde SC (2007). Lack of repeat transduction by recombinant adeno-associated virus type 5/5 vectors in the mouse airway. J. Virol..

[31] Bertin B, Veron P, Leborgne C, Deschamps JY, Moullec S, Fromes Y (2020). Capsid-specific removal of circulating antibodies to adeno-associated virus vectors. Sci. Rep..

[32] Elmore ZC, Oh DK, Simon KE, Fanous MM, and Asokan A. Rescuing AAV gene transfer from neutralizing antibodies with an IgG-degrading enzyme. *JCI Insight. ***5**(19), e139881 (2020). 10.1172/jci.insight.139881 PMC7566709 32941184

[33] Leborgne C, Barbon E, Alexander JM, Hanby H, Delignat S, Cohen DM (2020). IgG-cleaving endopeptidase enables in vivo gene therapy in the presence of anti-AAV neutralizing antibodies. Nat. Med..

[34] Hyde SC, Pringle IA, Abdullah S, Lawton AE, Davies LA, Varathalingam A (2008). CpG-free plasmids confer reduced inflammation and sustained pulmonary gene expression. Nat. Biotechnol..

[35] Meyer-Berg H, Zhou Yang L, Pilar de Lucas M, Zambrano A, Hyde SC, Gill DR (2020). Identification of AAV serotypes for lung gene therapy in human embryonic stem cell-derived lung organoids. Stem. Cell Res. Ther..

[36] Rose AC, Goddard CA, Colledge WH, Cheng SH, Gill DR, Hyde SC (2002). Optimisation of real-time quantitative RT-PCR for the evaluation of non-viral mediated gene transfer to the airways. Gene. Ther..

[37] Faul F, Erdfelder E, Lang AG, Buchner A (2007). G*Power 3: a flexible statistical power analysis program for the social, behavioral, and biomedical sciences. Behav. Res. Methods..

